# Severe Multisystem Inflammatory Symptoms in 2 Adults after Short Interval between COVID-19 and Subsequent Vaccination

**DOI:** 10.3201/eid2805.212316

**Published:** 2022-05

**Authors:** Elizabeth R. Jenny-Avital, Ruth A. Howe

**Affiliations:** Jacobi Medical Center, Bronx, New York, USA (E.R. Jenny-Avital);; Albert Einstein College of Medicine, Bronx (R.A. Howe)

**Keywords:** COVID-19, multisystem inflammatory syndrome in adults, MIS-A, coronavirus disease, SARS-CoV-2, severe acute respiratory syndrome coronavirus 2, viruses, respiratory infections, zoonoses, mRNA vaccine, United States

## Abstract

We observed multisystem inflammatory syndrome in 2 older adults in the United States who had received mRNA coronavirus disease vaccine soon after natural infection. We identified 5 similar cases from the Vaccine Adverse Events Reporting System. The timing of vaccination soon after natural infection might have an adverse effect on the occurrence of vaccine-related systemic inflammatory disorders.

The Centers for Disease Control and Prevention (CDC) recommends coronavirus disease (COVID-19) vaccination after natural severe acute respiratory syndrome coronavirus 2 (SARS-CoV-2) infection once acute symptoms resolve. We encountered 2 adults at Jacobi Medical Center (Bronx, NY, USA) who experienced severe febrile multisystem inflammatory illness, fulfilling the original CDC surveillance definition for multisystem inflammatory syndrome in adults (MIS-A) (*1*), after receiving COVID-19 mRNA vaccination 30 days after natural SARS-CoV-2 infection. We subsequently identified 5 similar cases from the Vaccine Adverse Event Reporting System (VAERS; https://www.vaers.hhs.gov) through October 2021 in hospitalized adults >30 years of age.

## The Cases

Case 1 was in a 48-year-old healthcare worker with type 2 diabetes, hypertension, and obesity (body mass index 55) who experienced sinus symptoms and loss of taste and smell in January 2021 concurrent with a positive SARS-CoV-2 PCR test. Thirty days later, she received the first dose of the mRNA-1273 COVID-19 vaccine (Moderna, https://www.moderna.com). The next day, she awoke with malaise, fever, and a localized pruritic rash. Symptoms, including worsening rash, fever (103°F), headache, loose stools, and disabling joint pain, progressed over 5 days. Physical examination revealed tachycardia (130 beat/min), fever (100.2°F), relative hypotension (100/60 mm Hg), swollen hands, and a rash consisting of urticarial pink papules and confluent red plaques involving her extremities and abdomen. Laboratory tests showed leukocytosis (16.5 × 103/µL, 77% neutrophils), acute liver injury (bilirubin 2 mg/dL, aspartate aminotransferase 120 U/L, alanine transaminase 248 U/L), and elevated C-reactive protein (187 mg/L), ferritin (558 mcg/L), and D-dimer (2,698 ng/mL). Nucleoprotein (NP) antibody testing was positive, substantiating previous SARS-CoV-2 infection. Results of imaging and serologic testing (viral hepatitis, HIV, parvovirus, autoimmune arthritis) were unrevealing. Echocardiography showed a small pericardial effusion. Treatment with prednisone and topical steroids resulted in rapid clinical improvement and resolution of her liver injury. Eleven days later, the palms of the patient’s hands and soles of her feet desquamated. After her second mRNA-1273 vaccine, she reported fever for 3 days. She had no symptoms after a booster with the BNT162b2 vaccine (Pfizer-BioNTech, https://www.pfizer.com).

Case 2 was in a healthy 51-year-old man who experienced self-limiting COVID-19 symptoms in mid-April 2021, concurrent with positive SARS-CoV-2 PCR tests in household contacts. He received the first dose of the mRNA BNT162b2 vaccine on May 11. Two weeks later, he experienced fever, watery diarrhea, and escalating abdominal discomfort. He sought care on May 31 for symptoms of fever (101.8°F) and diarrhea. He had tachycardia (130 beats/min), hypotension (90/60 mm Hg), leukocytosis (19.4 × 103/µL, 92% neutrophils), anemia (hemoglobin 11 g/dL), thrombocytopenia (72,000/µL), and elevated C-reactive protein (334 mg/L), Pro-Brain Natriuretic peptide (17,768 pg/mL), troponin (0.248 µg/L). NP antibody testing confirmed previous SARS-CoV-2 infection. PCR testing for SARS-CoV-2 and enteric pathogens was negative. Imaging of the chest and abdomen was initially normal. Despite fluids, he required vasopressors and overt pulmonary edema developed. Echocardiography confirmed biventricular dilatation with ejection fraction of 20%. After empiric MIS-A treatment with steroids and 1 dose of intravenous immunoglobulin (0.8 g/kg), symptoms, hemodynamics, and inflammatory markers rapidly improved; ejection fraction was normal (60%) on June 14 and June 28 while the patient was on prednisone (5 mg/d). On steroids, he experienced superficial desquamation of the palms of his hands and soles of his feet and 2 episodes of mild conjunctivitis. He remained fully recovered as of February 2022 but had no further vaccination.

We queried the VAERS database through October 2021 for hospitalized older adults (>30 years of age) using the symptom search term “Multisystem Inflammatory Syndrome/MIS” and found 19 cases (including case 2). VAERS did not substantiate MIS in 6 cases. Of the remaining cases, 3 additional cases occurred after a first vaccination given within 1 month of mild COVID-19 illness (Table). Only one other report provided information on previous COVID-19 (4 months earlier). Using search terms “myocarditis/fever” (57 cases) and “acute heart failure/fever” (12 cases), we found 1 case for each search that fulfilled criteria for MIS-A after vaccine administration soon after mild COVID-19 (Table).

**Table:** Characteristics of 5 previously published MIS cases occurring after COVID-19 vaccine was administered within 1 month of infection, United States*

Case no. and search term	VAERS ID	Patient age, y/sex	COVID-19 date	Vaccine date, type†	Description in VAERS	Treatment and outcome
1. MIS	1396536	53/F	2021 May 7	2021 May 29, Pfizer-BioNTech	2021 May 31: febrile (101.3°F), initial GI symptoms, dyspnea; admitted June 1; hypotensive (63/48 mm Hg) requiring vasopressors; leukocytes 31.3 × 10^3^ cells/µL, creatinine 4.6 mg/dL, bilirubin 5.5 mg/dL, EF 35%	Immunoglobulin infusion for prolonged hypotension despite antibiotics; weaned from vasopressors, reduced EF, and renal failure resolved
2. MIS	1282200	40/M	2020 Dec 26	2021 Jan 25, Pfizer-BioNTech	2021 Jan 29: fever, headache, neck pain, weakness, fatigue, diarrhea, abdominal pain; admitted after 2 emergency department visits with elevated cardiac inflammatory markers (BNP and troponin)	Steroids, with complete resolution
3. MIS	1154625	48/F	2021 Dec 31	2021 Jan 22, Moderna	2021 Feb 1: MIS with GI symptoms, rash, conjunctival injection, encephalopathy, elevated BNP	Immunoglobulin infusion, steroids, aspirin, with good response
4. Acute heart failure and fever	1027010	45/M	2020 Dec 30	2021 Jan 22, Pfizer-BioNTech	2021 Jan 30: fever, hypotension, morbilliform rash, cariogenic shock, EF 35%, CRP >320, BNP 3,583, SARS-CoV-2 antibody-positive	Intra-aortic balloon pump, antibiotics; resolution, with EF 67%
5. Myocarditis and fever	1088210, 1122743	46/F	2021 early Jan	2021 Feb 5‡	2021 Feb 23: 5 d fever, sore throat, swelling in hands/feet, EF 35%, hypotension requiring vasopressor, CRP >300 mg/L, ferritin 3,054 mcg/L, severe thrombocytopenia	Antibiotics, steroids, mechanical ventilation, ECMO, intra-aortic balloon pump support

*BNP, brain natriuretic peptide; COVID-19, coronavirus disease; CRP, C-reactive protein; ECMO, extracorporeal membrane oxygenation; EF, ejection fraction; FU, follow-up; GI, gastrointestinal; ID, identification; MIS, multisystem inflammatory syndrome; SARS-CoV-2, severe acute respiratory syndrome coronavirus 2; VAERS, Vaccine Adverse Event Reporting System. †Pfizer-BioNTech, https://www.pfizer.com; Moderna, https://www.moderna.com. ‡Vaccine type not available in VAERS report.

## Conclusions

Although case 1 fulfilled the initial 5-criteria surveillance CDC definition for MIS-A (*1*), which included acute liver injury, it does not fulfill the updated CDC definition (*2*), illustrating the dynamic and competing objectives of surveillance and precision. A broader Brighton Collaboration definition of MIS (*3*) was developed in part to be used in the evaluation of vaccine adverse events.

Case 2, by contrast, unequivocally fulfills MIS-A criteria and occurred within the usual time frame for post–COVID-19 MIS-A; its occurrence after vaccine might have been coincidental. Because MIS is overwhelmingly a disease of children and young adults, these 2 rare events, both occurring soon after vaccination in older adults, raised our concern that vaccination soon after COVID-19 infection might provoke MIS-A (case 2) or some similar vaccine-related multisystem inflammatory illness (case 1) consistent with the broader Brighton definition (*3*).

Some vaccine-triggered inflammatory symptoms, such as fever and myocarditis, occur disproportionately after a second vaccination, except in persons with previous COVID-19 infection, in whom reactions occur after a first vaccination, which suggests priming by a first antigenic exposure. The mRNA vaccine trials excluded participants with previous COVID-19, but antibody tests indicated previous infection in 2.5% of participants <65 years of age in the mRNA-1273 trial (*4*). Fever after first vaccination occurred in 9.4% of participants with previous COVID-19, compared with only 0.5% in COVID-19–naive participants and increased to 15.7% in the initially COVID-19–naive after the second vaccination (*4*). Similarly, myocarditis, a well-recognized vaccine adverse reaction in adolescents and young adults, almost invariably follows a second mRNA vaccine dose (*5*,*6*). However, a well-characterized report of 23 members of the US military identified myocarditis after the first vaccination only in 3 persons who had previous COVID-19 (*6*). By analogy, vaccine-associated multisystem inflammation, including MIS-A, might occur differentially between COVID-19–naive and COVID-19–experienced persons, such as suggested by the Brighton Collaboration document (*3*).

MIS, initially described in children who were SARS-CoV-2–negative by PCR but had plausible COVID-19 exposure or NP antibodies (*7*,*8*), was interpreted as a postviral syndrome caused by a deleterious hyper-inflammatory immune response (*7*). Although subsequent MIS cases reported in adults and children had concurrently positive PCR results in more than half (*1*,*9*,*10*), this finding was attributed to prolonged SARS-CoV-2 shedding, which has been noted in up to 19% of asymptomatic convalescent outpatients (*11*), rather than to a second infection in a sensitized host. Of 6 cases of MIS-A reported by Kaiser Permanente, 3 (50%) occurred in persons who were vaccinated after natural infection, despite the fact that only 7% of the cohort was vaccinated (*12*). Of 20 MIS-A cases collected by CDC during December 2020–April 2021, 7 (35%) occurred after vaccination after natural infection (*2*). The interval from infection to MIS-A was the same regardless of intervening vaccination, suggesting that vaccination was coincidental. Miyazato et al. (*13*) reported MIS-A 5 days after vaccination in a person with severe inflammatory illness that followed unrecognized previous COVID-19 infection confirmed only by positive NP antibody. Nune et al. (*14*) coined the term MIS-V to describe a case of MIS that began as progressive local injection-site inflammation 2 days after vaccination and demonstrated evolving systemic features, without evidence of antecedent COVID-19 infection.

COVID-19 vaccination during high periods of transmission increases the likelihood of vaccination following soon after infection. Further epidemiologic observations are needed to confirm a clear causal relationship, but our results indicate that vaccination soon after natural infection may result in the occurrence of strictly defined MIS-A or of other vaccine-triggered systemic inflammatory disorders.
